# Post-enrollment academic support and perceived learning effectiveness among international students in Chinese universities

**DOI:** 10.3389/fpsyg.2026.1937334

**Published:** 2026-09-30

**Authors:** Homa Bahmani, Yibin Ao, Jianjun Zhao, Abdussalam Khattak, Akeredolu Richard Joshua

**Affiliations:** 1 College of Environment and Civil Engineering, Chengdu University of Technology, Chengdu, China; 2 State Key Laboratory of Geohazard Prevention and Geo-Environment Protection, Chengdu University of Technology, Chengdu, China

**Keywords:** academic adaptation, Chinese higher education, educational psychology, international students, learning confidence, perceived academic support, perceived learning effectiveness, structural equation modeling

## Abstract

**Introduction:**

As international student mobility expands beyond traditional Anglophone destinations, universities increasingly need to understand how perceived academic support relates to students’ participation, adaptation, confidence, and learning effectiveness after enrollment. Less attention has been given to the relationship between academic support conditions and perceived learning effectiveness in non-Anglophone higher education contexts.

**Methods:**

This study tested an educational psychology framework linking selected background characteristics, perceived academic support conditions, and perceived learning effectiveness among 218 international students in Chinese universities. Data were analyzed using exploratory factor analysis, follow-up confirmatory factor analysis, and structural equation modeling with bootstrapping.

**Results:**

Student support services and academic quality, safety, and career preparedness showed significant positive associations with perceived learning effectiveness, whereas gender, funding type, and prior GPA showed no significant direct associations. Funding type was associated with academic and administrative reliability and learning environment quality but was not directly associated with perceived learning effectiveness.

**Discussion:**

The findings highlight the relevance of post-enrollment academic support conditions for international students’ perceived learning effectiveness, with implications for advising, communication support, counseling access, and career-related guidance.

## 1 Introduction

International students’ mobility is increasingly shaped by destinations beyond the traditional Anglophone receiving countries. This transition is driven by affordability, geopolitical dynamics, and the rapid rise in the global ranking and capacity of Asian higher education systems (ICEF Monitor, 2024). China, in particular, has emerged as a major host country due to sustained policy support, including the Belt and Road Initiative and targeted internationalization strategies (Ahmad and Shah, 2018; Yuan et al., 2021; Yue et al., 2024). Since the reform and opening-up period beginning in 1978 (Ma and Zhao, 2018), China has gradually expanded international student education and positioned itself as an important global study destination (Ding, 2016; Hussain and Shen, 2019). This policy support is reflected in broader educational research, which underscores China’s strategic efforts to attract international students through programs and infrastructure improvements (Wang et al., 2024). However, the expansion of international enrollment raises a second and increasingly important question: what happens after students arrive? Internationalization cannot be evaluated solely by recruitment numbers, institutional visibility, or scholarship expansion; it must also be assessed by the academic support conditions that enable students to participate, communicate, adapt, and perceive their learning as effective after enrollment.

This question is especially important in non-Anglophone higher education contexts. Although international students may be attracted by affordability, academic opportunities, institutional reputation, and access to scholarships, they often encounter additional challenges related to language, academic communication, administrative navigation, teaching styles, and limited integration with domestic student communities. Therefore, post-enrollment academic support is not a peripheral service issue. It is part of the learning environment through which international students access information, interact with faculty and staff, understand academic expectations, seek assistance, and prepare for future academic or career pathways. From an educational psychology perspective, such support conditions may shape not only satisfaction, but also engagement, confidence, participation, and perceived learning effectiveness. In this study, academic support is treated as an educational psychology condition rather than only an institutional service. The central assumption is that students’ perceptions of support may influence learning effectiveness through academic participation, access to communication, learning confidence, and adaptation to the host educational environment. This framing is particularly relevant for international students, whose learning perceptions may depend on how they interpret and use available support within unfamiliar academic and linguistic systems.

Recent research has increasingly shifted from international student recruitment to the quality of students’ post-enrollment experiences, including academic support, institutional communication, adaptation, satisfaction, and learning outcomes (Ammigan et al., 2021; Hao et al., 2024; Martirosyan et al., 2019). Within this literature, three thematic strands are particularly relevant.

First, studies of higher education perceived academic support conditions have shown that institutional responsiveness, academic support, administrative reliability, and infrastructure are associated with student satisfaction and perceived institutional value. Traditional models such as SERVQUAL and HEdPERF have been widely adopted in higher education contexts to examine tangibles, reliability, responsiveness, assurance, empathy, and broader service-quality dimensions (Abdullah, 2006; Yousapronpaiboon, 2014). More recent studies extend these models by incorporating student-centered and digital learning environments, support services, institutional communication, and administrative responsiveness (Ali et al., 2018; Bouranta et al., 2024; Dalban et al., 2025; Hoque et al., 2023; Surya Bahadur et al., 2024; Vinueza-Martínez, 2026). However, many studies in this stream stop at satisfaction or perceived value, rather than linking support conditions to students’ perceived learning effectiveness.

Second, research on learning effectiveness has increasingly moved beyond institution-level performance indicators and formal academic achievement measures. Educational effectiveness has often been examined through macro-level efficiency models, institutional indicators, or student achievement outcomes (Giambona et al., 2011; Guo and Ye, 2025; Perelman and Santin, 2011). On the other hand, student-centered approaches argue that learning effectiveness should also be examined through students’ perceptions of academic development, engagement, communication improvement, confidence, and ability to apply knowledge within the available learning environment (Dima et al., 2024; Hao et al., 2024; Mendoza-Villafaina and López-Mosquera, 2024; Xu et al., 2023). This perspective is particularly relevant for international students because perceived learning may be shaped not only by classroom instruction and prior academic ability, but also by whether the host institution provides accessible academic guidance, communication channels, advising, counseling, and career-relevant preparation. Recent evidence from Chinese educational settings further shows that students’ perceived support is associated with engagement, learning attitudes, and anxiety, reinforcing the importance of support conditions in shaping learning-related experiences (Zhou et al., 2025).

Third, studies on international students in non-Anglophone or non-Western higher education contexts have emphasized the role of language, culture, institutional responsiveness, and social integration in shaping educational experience. Existing research in China suggests that while international students may value infrastructure, affordability, and educational opportunity, they also face challenges related to academic communication, teaching styles, institutional procedures, and integration with domestic students (Ding, 2016; Ding et al., 2025; Wang and Lin, 2019). Similarly, broader research on international student adaptation indicates that language barriers, cultural distance, access to support, and opportunities for interaction influence students’ engagement and educational experiences in host countries (Alemu and Cordier, 2017; Bianchi, 2024; Gebregergis and Csukonyi, 2025). Nevertheless, these challenges should not be interpreted only as individual adaptation problems. They also reflect institutional design questions, including how universities organize academic support, communication assistance, advising, counseling, access to administrative responsiveness, and career-related guidance for students learning in unfamiliar educational and linguistic environments.

These strands address complementary parts of the international student experience but are rarely connected within one analytical framework. SERVQUAL-informed higher education models provide a structured way to examine the institutional conditions students encounter, including reliability, responsiveness, support, infrastructure, and academic or administrative provision. Learning-evaluation perspectives address a different question: how students evaluate the educational consequences of their experience beyond satisfaction alone. Kirkpatrick’s framework, although originally developed for training evaluation, has been adapted to higher education to organize educational effectiveness across Reaction, Learning, Behavior, and Result (Praslova, 2010). At the same time, applying it in higher education requires attention to contextual conditions rather than assuming the four levels operate as a fixed causal sequence (Cahapay, 2021). Linking these perspectives therefore provides a basis for examining whether the institutional conditions students encounter after enrollment are associated with how they evaluate their learning development, application, confidence, and future preparedness.

Despite these advances, important limitations remain. First, studies examining learning effectiveness often focus on institutional or macro-level indicators, while giving comparatively limited attention to students’ subjective learning experiences in international higher education contexts. Second, research on international students in China has extensively examined adaptation challenges, language barriers, satisfaction, and cross-cultural experience, but has less often connected these experiences to perceived learning effectiveness within a unified analytical framework. Third, although service-quality and institutional support studies identify important dimensions of student experience, they often treat satisfaction as the outcome rather than examining how post-enrollment support conditions relate to perceived learning effectiveness. As a result, less is known about whether international students’ perceived learning effectiveness is shaped more by who students are before enrollment or by the academic support conditions they encounter after enrollment.

To address these gaps, this study develops and tests a framework linking perceived academic support dimensions, selected student background characteristics, and perceived learning effectiveness among international students in Chinese higher education. Rather than treating international students’ learning experiences primarily as problems of individual adaptation, the study examines how post-enrollment academic support conditions, including student support services, academic and administrative reliability, learning environment and infrastructure, and academic quality, safety, and career preparedness, are associated with perceived learning effectiveness. The study uses survey data from international students in Chinese universities and applies exploratory factor analysis, confirmatory factor analysis, and structural equation modeling to examine the relationships among background characteristics, perceived academic support dimensions, and perceived learning effectiveness.

This study contributes to educational psychology and international higher education research in three ways. First, it shifts attention from recruitment-oriented internationalization to post-enrollment academic support conditions. Second, it positions student support services as part of the learning environment rather than as peripheral administrative provision. Third, it connects SERVQUAL-informed institutional support dimensions with a Kirkpatrick-informed conception of perceived learning effectiveness, allowing the study to examine associations between post-enrollment support conditions, selected background characteristics, and students’ perceived learning effectiveness. Although the empirical setting is China, the study speaks to a broader question relevant to diverse, non-Anglophone higher education systems: how institutions can design support, communication, advising, and career-preparation structures that enable international students to participate meaningfully and perceive their learning as effective.

The remainder of this paper is organized as follows. The next section presents the conceptual framework linking post-enrollment academic support conditions and perceived learning effectiveness. The Methods section describes the participants, measures, and analytical strategy. The Results section presents the measurement model, the structural model, and the indirect-effect analysis. The Discussion interprets the findings in relation to academic support, perceived learning effectiveness, and international student education in non-Anglophone higher education contexts, followed by implications, limitations, and future research directions.

## 2 Materials and methods

### 2.1 Conceptual framework and model development

Building on the research gap identified above, this study develops a framework linking selected student background characteristics, post-enrollment academic support conditions, and perceived learning effectiveness. The framework is based on the assumption that international students’ learning perceptions are shaped not only by who they are before or upon enrollment, but also by the support conditions they encounter within the host university. Therefore, the model distinguishes between two explanatory layers: background characteristics and perceived academic support dimensions.

The first layer includes selected background characteristics that may influence students’ educational experiences, such as gender, prior academic performance, funding type, country or region of prior education, and language proficiency. These variables were included because previous studies suggest that student background may shape adaptation, support needs, and academic participation among international students (Al Juboori et al., 2025; Azram et al., 2025). However, in the present framework, these characteristics are not considered sufficient explanations of perceived learning effectiveness. Rather, they are included to examine whether perceived learning effectiveness is more strongly associated with pre-enrollment characteristics or with the academic support conditions students experience after enrollment.

The second layer includes Institutional Support Dimensions (ISD), which represent students’ perceptions of post-enrollment academic support conditions in Chinese universities. The ISD framework was informed by SERVQUAL-based higher education models, but it does not frame students as customers. Instead, it provides a structured way to organize institutional conditions that may affect the learning experience, including physical and technological infrastructure, academic and administrative reliability, student support and communication, faculty competence and institutional credibility, and student-centered support services (Abdullah, 2006; Yousapronpaiboon, 2014). In this study, these dimensions are understood as parts of the learning environment through which international students access resources, communicate with institutional actors, understand academic expectations, and locate support when difficulties arise. The proposed framework assumes that ISD may influence perceived learning effectiveness by shaping students’ academic engagement, communication experiences, learning accessibility, and adaptation to the educational environment. These dimensions were expected to shape perceived learning effectiveness by influencing students’ academic participation, access to resources, communication with institutional actors, confidence, and adaptation to the learning environment.

Perceived learning effectiveness (PLE) is the framework’s outcome construct. Drawing on Kirkpatrick’s learning-evaluation perspective, PLE is conceptualized as students’ evaluation of their own learning experience rather than as an objective measure of academic achievement (Praslova, 2010). It includes perceived satisfaction with learning, academic development, knowledge acquisition, communication improvement, ability to apply knowledge, adaptation to academic environments, confidence, educational value, and future preparedness. This definition is important because the study examines students’ perceived learning development within a specific institutional support environment, not formal grades or longitudinal learning gains. In this layer, Reaction reflects students’ satisfaction with learning experiences and institutional support; Learning refers to perceived academic development, knowledge acquisition, and communication improvement; Behavior represents students’ perceived ability to apply knowledge, adapt to academic environments, and engage in educational activities; and Results capture broader perceptions of educational value, confidence development, and future preparedness. Although Kirkpatrick’s framework was originally developed for organizational training evaluation, it has subsequently been adapted to higher education as a structured approach to organizing learning outcomes and program effectiveness (Praslova, 2010). Its use here therefore does not imply that university education is equivalent to workplace training. Rather, the framework provides a way to conceptualize students’ educational experience beyond satisfaction alone by incorporating perceived learning, application, and broader educational consequences. Previous work has also cautioned against treating the four levels as a rigid causal hierarchy when the framework is applied in higher education and has emphasized the need to incorporate contextual conditions into evaluation (Cahapay, 2021). Accordingly, this study used Reaction, Learning, Behavior, and Results as conceptual categories for organizing the 13 PLE indicators rather than as separate or sequential latent subscales; PLE was modeled as one overarching latent construct.

The connection between ISD and PLE is therefore based on a distinction between institutional context and perceived educational effectiveness. SERVQUAL-informed dimensions identify conditions through which international students encounter academic and administrative procedures, support and communication, learning resources, safety, and career-related preparation. These conditions do not constitute learning outcomes themselves, but they can influence students’ opportunities to participate in, navigate, and use the educational environment. Academic and administrative reliability may reduce uncertainty in accessing academic processes; student support services may facilitate communication, guidance, and help-seeking; learning environment and infrastructure may support access to learning activities and resources; and academic quality, safety, and career preparedness may influence the perceived relevance and usability of the educational experience. These pathways provide the theoretical basis for expecting institutional support dimensions to be associated with the overarching PLE construct, which captures students’ perceptions of satisfaction, learning development, application, confidence, and future preparedness. The proposed framework therefore links the institutional conditions students experience after enrollment with their evaluation of learning effectiveness without assuming either causal relationships among Kirkpatrick’s four categories or causal effects from institutional support to learning. Figure 1 presents the conceptual framework guiding the study. The model links selected background characteristics, post-enrollment academic support conditions, and perceived learning effectiveness, thereby allowing the study to test whether international students’ perceived learning effectiveness is better explained by background characteristics or by the institutional support conditions they encounter after enrollment.

**Figure 1:**
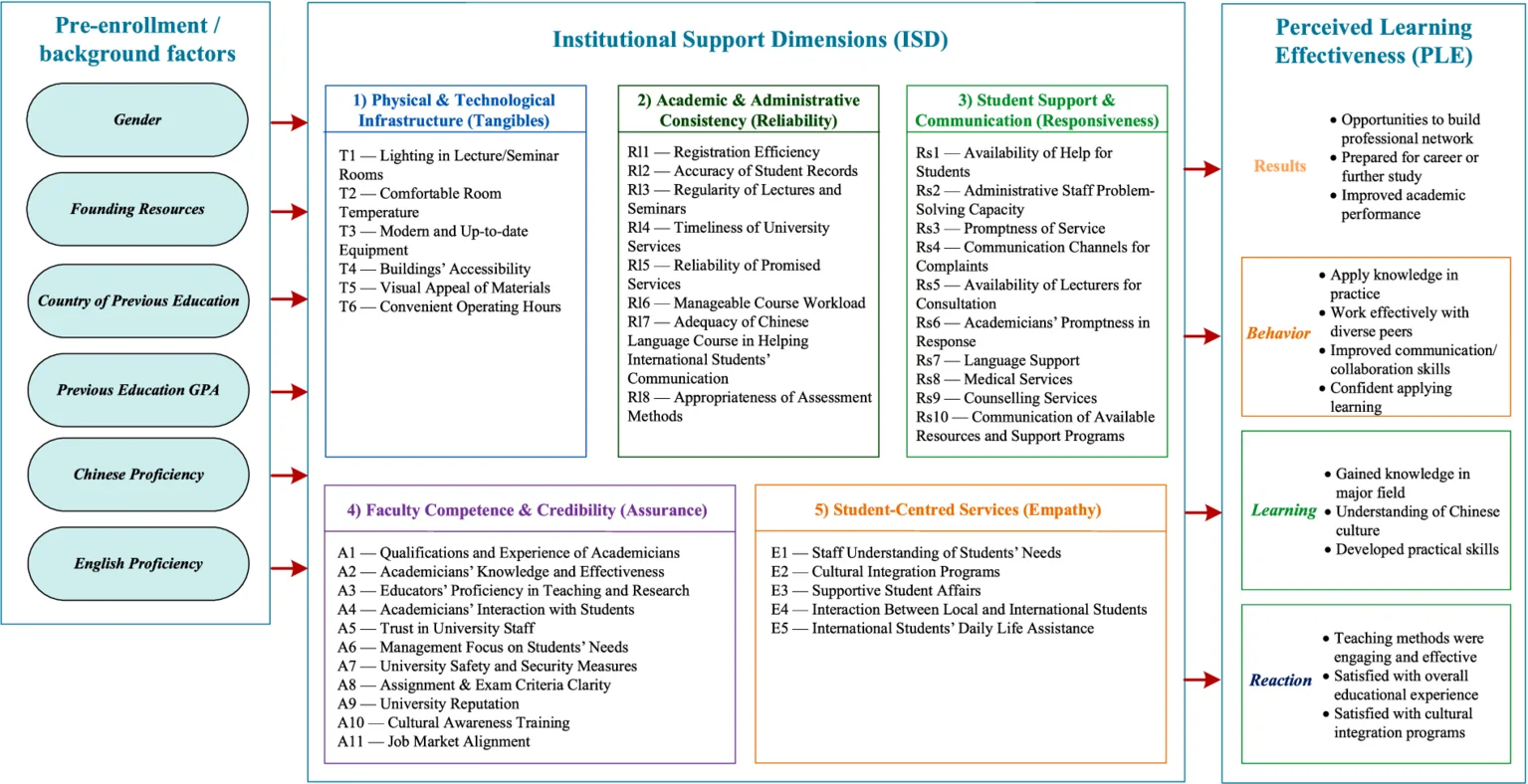
Conceptual framework linking post-enrollment support conditions and perceived learning effectiveness among international students. Reaction, Learning, Behavior, and Results represent conceptual groupings of the PLE indicators and were not modeled as separate latent factors.

### 2.2 Study context and research design

China provides a relevant context for examining international students’ post-enrollment academic support because it has become an important non-Anglophone host country for international higher education. Although international student recruitment and scholarship expansion have received considerable attention, less is known about how students experience academic support, communication, administrative reliability, learning infrastructure, safety, and career-related preparation after enrollment. This context is therefore suitable for examining whether international students’ perceived learning effectiveness is associated more closely with selected background characteristics or with the institutional support conditions they encounter within the host university environment.

This study used a cross-sectional, survey-based research design. The design was appropriate because the objective was not to measure academic achievement or longitudinal learning gain, but to examine students’ perceptions of institutional support conditions and perceived learning effectiveness at a single point in time. The analysis combined exploratory and confirmatory factor analyses and structural equation modeling to identify perceived academic support dimensions and test their relationships with perceived learning effectiveness. This sequence allowed the study to move from indicator reduction and preliminary assessment of the measurement structure to structural testing of the proposed model.

### 2.3 Participants and data collection

Data were collected through an online questionnaire administered to international students enrolled in Chinese higher education institutions. The survey was conducted from April 10 to May 21, 2025. Eligible respondents were international students enrolled in Chinese universities during the survey period who were able to complete the questionnaire voluntarily. The survey was designed to collect information on students’ background characteristics, perceived institutional support conditions, and perceived learning effectiveness.

A total of 330 responses were received. Responses were screened for completeness and validity before analysis. Incomplete or invalid responses were removed, resulting in 218 fully completed questionnaires for the final analysis. The final sample included international students enrolled in universities across several Chinese provincial-level regions, including Sichuan, Shaanxi, Guangdong, Guangxi, Jiangsu, Yunnan, Tianjin, Beijing, Hunan, Jiangxi, and Hubei.

The sample should be interpreted as an exploratory multi-institutional sample rather than a statistically representative sample of all international students in China. Although participants came from multiple universities and regions, the distribution was uneven across nationality, institution, and funding type. This limitation is important because international students in China are not a homogeneous group, and their learning experiences may differ by university type, discipline, funding arrangement, region, language environment, and level of institutional support.

Participation was voluntary, and informed consent was obtained before respondents completed the survey. The questionnaire was anonymous and did not collect personally identifiable information. The study received institutional ethics approval from the authors’ university. For peer review, the institution name is masked where required. Quality-control procedures included screening for incomplete or invalid responses, retaining only fully completed questionnaires for analysis, and evaluating internal consistency before testing the structural model.

### 2.4 Variables and measures

The questionnaire included three main sections. The first section collected background characteristics, including gender, funding type, prior education GPA, country or region of previous education, and language proficiency. These variables were included because previous research suggests that students’ pre-enrollment characteristics and educational backgrounds may shape adaptation, support needs, and academic experience among international students. In the final structural model, gender, funding type, and prior education GPA were included as observed background predictors because they were theoretically relevant and supported by the model-development process.

The second section measured Institutional Support Dimensions (ISD). The ISD items were developed from a SERVQUAL-informed higher education framework and adapted to the international student context in China. In this study, ISD refers to students’ perceptions of the post-enrollment academic support conditions they encounter within their host institutions. These conditions included physical and technological infrastructure, academic and administrative reliability, student support and communication, faculty competence and institutional credibility, and student-centered support services. The ISD framework was not used to position students as customers. Rather, it was used as an organizing structure to examine the institutional conditions through which students access academic information, communicate with staff and faculty, use learning resources, seek support, and prepare for academic or career development.

The third section measured perceived learning effectiveness (PLE). PLE was adapted from Kirkpatrick’s learning-evaluation perspective and reflected students’ perceived satisfaction with learning experiences, academic development, knowledge acquisition, communication improvement, ability to apply knowledge, academic adaptation, confidence, educational value, and future preparedness. In the final measurement model, PLE was represented by 13 indicators organized conceptually around Reaction, Learning, Behavior, and Results. These categories were used to structure the content of PLE rather than specified as separate latent subscales. PLE was therefore treated as a student-reported learning perception rather than an objective measure of academic achievement. All ISD and PLE items were measured using a five-point Likert scale. The ISD item pool was primarily grounded in previously published higher-education service-quality measures, with item-level sources reported in Supplementary Table 1. Among the 17 indicators retained following exploratory analysis, 13 were adapted from published measures, while four context-specific indicators addressed language support, adequacy of Chinese-language provision, job-market alignment, and interaction between local and international students. The instrument is therefore treated as a literature-informed, context-adapted measure rather than as a newly developed and independently content-validated scale.

### 2.5 Analytic strategy

The analysis proceeded in four stages. First, the data were screened for completeness and response validity. Descriptive statistics were then used to summarize participants’ demographic and educational characteristics. This step provided the empirical profile of the sample and clarified the background conditions under which students evaluated their institutional support and learning experiences.

Second, Kruskal–Wallis tests were used as an exploratory diagnostic procedure to examine whether theoretically derived ISD indicators varied across selected demographic and educational background groups. These tests were not treated as the primary basis for item retention or factor-structure determination. Instead, they were used to support preliminary screening and to identify indicators that showed variation across student groups. Final item retention was based on theoretical relevance, factor loadings, cross-loadings, internal consistency, and the interpretability of the latent constructs. For the Kruskal–Wallis analysis, countries were grouped into South Asia, Southeast Asia, Sub-Saharan Africa, the Middle East and North Africa, East Asia, and Western countries. Kruskal–Wallis results were used solely for exploratory screening and are reported separately for transparency. The final construct structure was determined based on theoretical relevance, factor loadings, cross-loadings, internal consistency, and interpretability, rather than on group-difference significance alone.

Third, an exploratory factor analysis using principal component extraction and varimax rotation was conducted to reduce the ISD items into a set of constructs. Sampling adequacy was assessed using the Kaiser–Meyer–Olkin measure, and Bartlett’s Test of Sphericity was used to examine whether the correlation matrix was suitable for factor analysis. The KMO value was 0.875, indicating strong sampling adequacy, and Bartlett’s Test of Sphericity was significant, *χ*^2^(153) = 1320.415, *p* < 0.001. The exploratory analysis identified four institutional support dimensions: Academic and Administrative Reliability, Student Support Services, Learning Environment and Infrastructure, and Academic Quality, Safety, and Career Preparedness. Together, these dimensions explained 60.07% of the variance. Internal consistency was assessed using Cronbach’s alpha, with all retained dimensions exceeding the 0.70 threshold. Table 1 presents the EFA-derived latent variables, the retained ISD indicators, the factor loadings, and the reliability coefficients.

**Table 1:** EFA-derived latent variables, retained ISD indicators, and factor loadings.

Component name	ISD Indicators	Loadings
Academic and Administrative (AAR) (Cronbach’s alpha = 0.81)	Rl4	0.851
	Rl3	0.779
	Rl2	0.687
	Rl5	0.612
Student Support Services (SSS) (Cronbach’s alpha = 0.738)	Rs7	0.832
	Rl7	0.723
	Rs9	0.644
	Rl6	0.525
Learning Environment and Infrastructure (LEI) (Cronbach’s alpha = 0.794)	T2	0.802
	T3	0.730
	T1	0.698
	T4	0.547
	T5	0.521
Academic Quality, Safety, and Career Preparedness (AQSCP) (Cronbach’s alpha = 0.702)	A3	0.755
	A7	0.707
	A11	0.649
	E4	0.549

Fourth, a follow-up confirmatory factor analysis was conducted in AMOS 24 to assess the compatibility of the EFA-derived measurement structure with the observed data. Because the EFA and CFA were conducted using the same analytical sample, the CFA was treated as a preliminary confirmatory assessment rather than as an independent validation of the factor structure. Model fit was evaluated using multiple indices, including CMIN/DF, RMSEA, RMR, CFI, and TLI. Because fit indices may be sensitive to sample size, model complexity, and context-specific measurement structures, model fit was interpreted using a combination of absolute, incremental, and parsimony-oriented indices rather than relying on a single threshold. Additional measurement properties of the ISD constructs were examined using standardized CFA loadings and composite reliability (CR). Discriminant validity among the four ISD dimensions was assessed using the heterotrait–monotrait ratio (HTMT), with 0.85 and 0.90 used as reference criteria. Because the focal ISD and PLE constructs were measured from the same respondents within the same survey, potential common method variance was additionally examined using a post-hoc single-factor CFA. All 30 retained ISD and PLE indicators were specified to load on one latent factor to examine whether a single common factor could plausibly account for the covariance among the self-reported measures. This diagnostic was used to assess the presence of a dominant common factor rather than to rule out common method bias entirely.

Modification indices (MIs) and expected parameter changes (EPCs) were also examined as diagnostic indicators of localized model misspecification. The final measurement specification included residual covariances only among indicators assigned to the same latent construct, thereby allowing localized residual dependence among conceptually related indicators without introducing cross-construct residual associations. Remaining MIs were examined for potential cross-loadings and cross-construct residual relationships. We did not introduce additional cross-construct parameters solely to improve global fit because such post-hoc modifications could increase the risk of sample-specific overfitting. Details of the retained residual covariance structure and the largest remaining modification indices are provided in the Supplementary material.

After the measurement model was assessed, structural equation modeling was used to examine relationships among selected background characteristics, ISD, and perceived learning effectiveness. The structural model tested three sets of relationships: first, whether selected background characteristics were directly associated with PLE; second, whether these characteristics were associated with students’ perceptions of ISD; and third, whether statistically significant indirect associations with PLE were present through ISD. Bootstrapping with 2,000 samples and bias-corrected 95% confidence intervals was used to assess indirect effects.

The final SEM included gender, funding type, and prior education GPA as observed background predictors. ISD and PLE were specified as endogenous constructs. This structure allowed the study to examine the pattern of direct and indirect associations among selected background characteristics, post-enrollment institutional support dimensions, and perceived learning effectiveness. Figure 2 presents the hypothesized structural model tested in the SEM.

**Figure 2:**
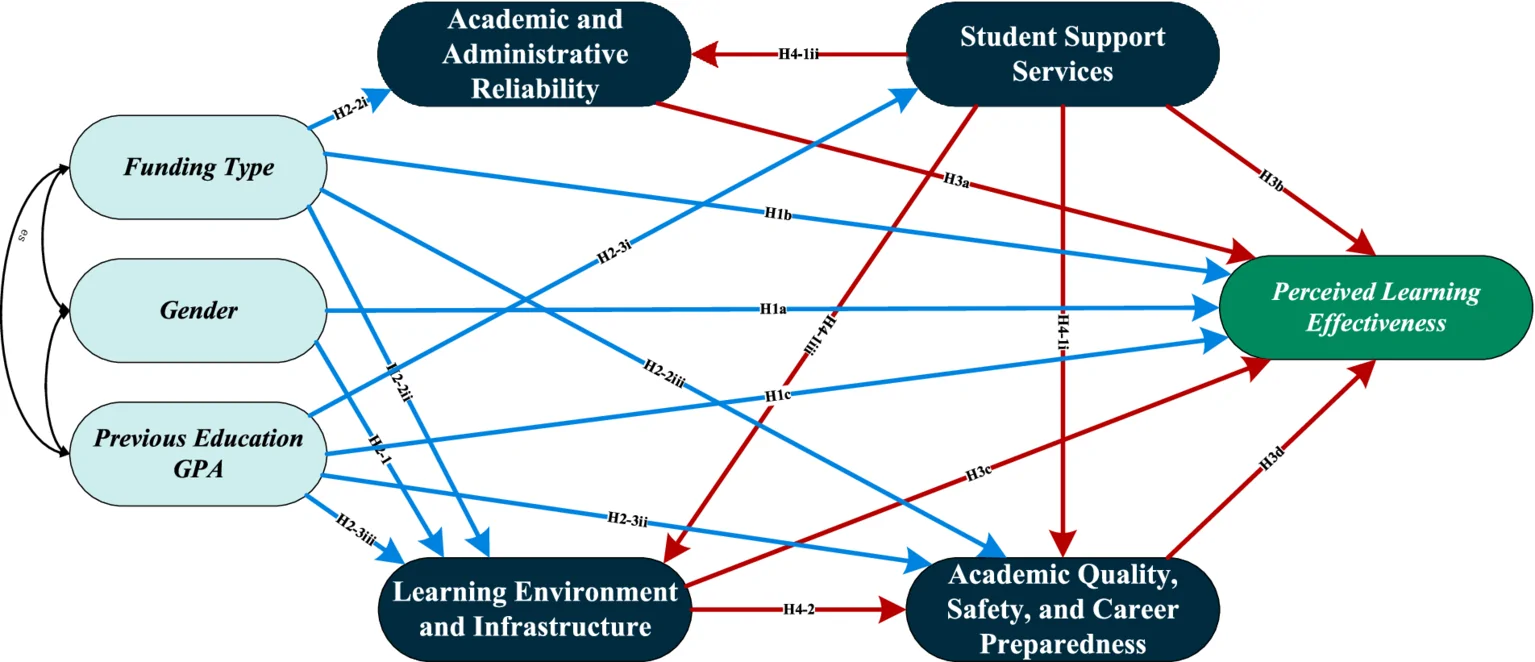
Hypothesized structural model tested in the SEM.

## 3 Results

### 3.1 Participant characteristics and measurement model

The sample included international students with sufficient exposure to the Chinese higher education context to evaluate their academic and institutional experiences. Only 1.8% of respondents had resided in China for less than 6 months. Respondents represented 39 nationalities, although the distribution was uneven, with the largest groups from Pakistan (32.6%) and Nigeria (12.8%). Male students represented 62.8% of the sample. Most respondents were graduate students: 51.8% were enrolled in master’s programs and 29.4% in doctoral programs. The average age was 28.26 years (SD = 5.19), and the mean prior GPA was 3.42 on a 4.0 scale (SD = 0.38). Regarding funding, 54.6% received university scholarships, 31.2% received Chinese Government Scholarships, and 6.0% were self-supported. English proficiency was relatively high, whereas Chinese proficiency was more limited: 59.6% reported native or advanced English proficiency, while only 13.8% reported native or advanced Chinese proficiency and 20.2% reported no Chinese proficiency.

A confirmatory factor analysis was conducted in AMOS 24 to assess the EFA-derived latent structure comprising Academic and Administrative Reliability (AAR), Student Support Services (SSS), Learning Environment and Infrastructure (LEI), Academic Quality, Safety, and Career Preparedness (AQSCP), and perceived learning effectiveness (PLE). The measurement model demonstrated mixed fit. The CMIN/DF value was 1.737, while RMSEA (0.058) and RMR (0.041) indicated acceptable absolute and approximate fit. However, CFI (0.892) and TLI (0.870) were below the conventional 0.90 threshold, indicating marginal incremental fit. Taken together, these indices provide preliminary rather than definitive support for the proposed measurement structure.

Additional measurement checks were conducted for the four ISD dimensions. The standardized CFA loadings of the retained ISD indicators ranged from 0.525 to 0.770, with all loadings above 0.50. Composite reliability was 0.803 for AAR, 0.722 for SSS, 0.767 for LEI, and 0.678 for AQSCP. AAR, SSS, and LEI therefore exceeded the 0.70 criterion, while AQSCP was slightly below this level. Discriminant validity was examined using HTMT, with values ranging from 0.567 to 0.670. All values remained below both the 0.85 and 0.90 reference criteria, indicating adequate distinction among AAR, SSS, LEI, and AQSCP (Supplementary Table 7). A post-hoc single-factor CFA was also conducted to examine whether one common factor could account for the covariance among the retained ISD and PLE indicators. The model showed poor fit, CMIN/DF = 3.72, RMSEA = 0.112, CFI = 0.629, and TLI = 0.602, substantially poorer than the proposed multidimensional measurement model (Supplementary Table 4). This finding does not support a single dominant common factor as an adequate explanation of the covariance among the measures, although partial common method variance cannot be excluded.

Examination of modification indices indicated localized residual overlap rather than a single dominant source of misspecification. The final measurement specification contained 31 within-construct residual covariances, of which 21 involved PLE indicators, and 10 were distributed across the four institutional support constructs. After accounting for these within-construct residual relationships, eight remaining covariance modification indices exceeded 10. The largest involved counseling services and local–international student interaction (MI = 15.68), job-market alignment and satisfaction with cultural integration programs (MI = 15.46), and satisfaction with cultural integration programs and the SSS construct (MI = 12.67). In contrast, no suggested additional regression weight or cross-loading had an MI of 10 or greater; the largest was 7.44. Because the larger remaining modification indices predominantly implied relationships across the specified construct boundaries, these parameters were not added solely to improve model fit. This pattern suggests some localized conceptual overlap among support, cultural-integration, and perceived-learning indicators and supports cautious interpretation of the measurement structure (Supplementary Table 5).

### 3.2 Structural model results

Following the preliminary assessment of the measurement model, SEM was used to test the hypothesized relationships among selected student background characteristics, ISD, and PLE. In the structural model, gender, funding type, and previous education GPA were specified as observed exogenous variables. The ISD and PLE were specified as endogenous variables in accordance with the proposed analytical framework.

Next, the overall fit of the structural model was evaluated using multiple fit indices. The structural model showed a fit pattern broadly comparable to the measurement model. The CMIN/DF increased only slightly from 1.737 in the measurement model to 1.766 in the structural model, remaining below the recommended threshold of 2.0. RMSEA also increased only minimally from 0.058 to 0.059, remaining within the commonly accepted range for good approximate fit. RMR remained unchanged at 0.041, below the 0.05 threshold. However, the incremental fit indices were marginal: CFI decreased from 0.892 to 0.885, and TLI decreased from 0.870 to 0.865, both falling slightly below the conventional 0.90 criterion. Rather, the combination of acceptable absolute fit and marginal incremental fit provided a basis for cautious, provisional examination of the hypothesized structural relationships.

The structural model was tested to evaluate the hypothesized relationships among student characteristics, ISD, and PLE. To test the significance of indirect effects, bootstrapping with 2,000 samples was performed. Bias-corrected 95% confidence intervals were used, as this method provides more accurate estimates in the presence of non-normal sampling distributions. The results of the SEM (shown in Table 2 and Figure 3) provided partial support for the hypothesized relationships. Regarding background characteristics, no significant direct effects were observed on students’ perceptions of learning effectiveness. Specifically, gender, funding type, and prior education GPA all failed to demonstrate statistically meaningful associations with learning effectiveness perception, therefore providing no support for Hypothesis 1. Within the tested model, gender, funding type, and prior GPA did not show significant direct associations with PLE, whereas selected institutional support dimensions did. This pattern should not be interpreted as evidence that the background characteristics are unimportant or that the institutional associations are necessarily stronger.

**Table 2:** Results of structural path analysis.

Path	Estimate	*p*-value	Interpretation
Funding type ↔ Prior Education GPA	0.187	0.015	Significant correlation
Gender → PLE	0.048	0.410	H1a: Rejected
Funding type → PLE	−0.002	0.987	H1b: Rejected
Prior Education GPA → PLE	−0.004	0.886	H1c: Rejected
Gender → LEI	−0.024	0.777	H2-1: Rejected
Funding type → AAR	−0.139	0.003	H2-2i: Accepted
Funding type → LEI	−0.168	0.013	H2-2ii: Accepted
Funding type → AQSCP	−0.091	0.193	H2-2iii: Rejected
Prior Education GPA → SSS	0.104	0.319	H2-3i: Rejected
Prior Education GPA → AQSCP	−0.023	0.85	H2-3ii: Rejected
Prior Education GPA → LEI	0.066	0.359	H2-3iii: Rejected
AAR → PLE	−0.267	0.142	H3a: Rejected
SSS → PLE	0.797	< 0.001	H3b: Accepted
LEI → PLE	−0.138	0.774	H3c: Rejected
AQSCP → PLE	1.158	0.009	H3d: Accepted
SSS → AQSCP	1.085	< 0.001	H 4–1i: Accepted
SSS → AAR	0.793	< 0.001	H 4–1ii: Accepted
SSS → LEI	0.797	< 0.001	H 4–1iii: Accepted
LEI → AQSCP	−0.288	0.318	H 4–2: Rejected

**Figure 3:**
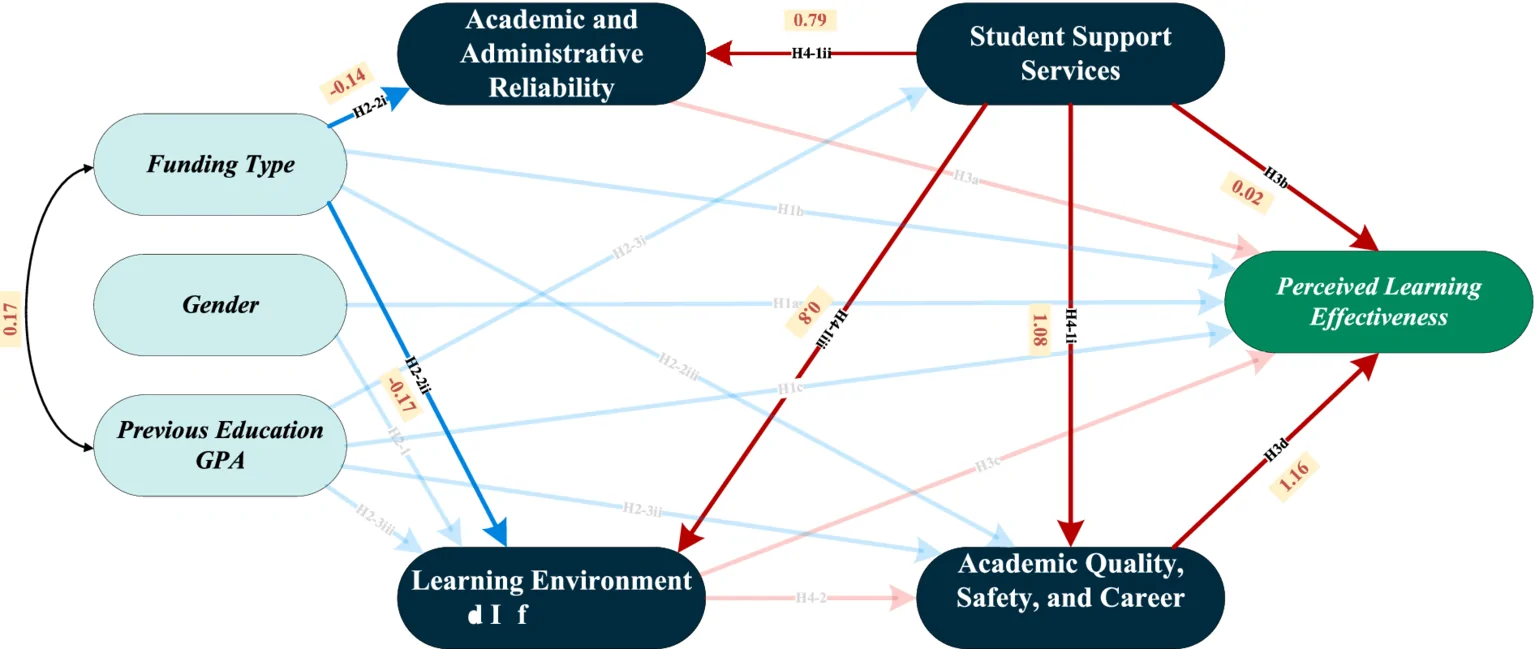
Structural model results for institutional support dimensions and perceived learning effectiveness.

Similarly, Hypothesis 2–1, which posited that gender would influence perceptions of student support services and the learning environment, was not supported by the data. While gender showed no significant relationship with either construct, a notable finding emerged under the first two Hypotheses of 2–2: funding type was negatively associated with perceptions of academic and administrative reliability and the physical facilities of the learning environment. The negative path coefficients from funding type to academic and administrative reliability (*β* = −0.139) and learning environment (*β* = −0.168) suggest that students with partial or self-sourced funding tend to report lower perceptions of academic and administrative reliability and learning conditions. However, no significant effect was found between funding type and perceptions of academic quality, safety, and career preparedness, partially supporting this hypothesis. No statistically significant associations were found between prior GPA and the institutional-level constructs; therefore, Hypotheses 2 and 3 were not supported. The lack of significant associations involving prior GPA suggests that prior academic performance alone was not strongly associated with students’ perceptions of institutional quality or learning effectiveness within this sample.

In contrast, the tested model provided statistical support for two institutional-level associations with perceived learning effectiveness. Student support services showed a statistically significant positive association with students’ PLE, and academic quality, safety, and career preparedness were also significantly associated with PLE, supporting Hypotheses 3b and 3d within the specified model. Academic and administrative reliability and learning environment were not significantly associated with PLE when the other institutional dimensions were included. These results indicate that academic quality, safety, and career preparedness and support services showed significant associations with PLE within the specified model, whereas independent direct associations were not detected for academic and administrative and learning environment and infrastructure. Given the marginal incremental fit of the measurement and structural models, these relationships should be interpreted cautiously.

The tested model also provided statistical support for Hypothesis 4–1. Student support services were significantly associated with academic quality and career preparation, academic and administrative reliability, and the learning environment. This broader pattern of associations suggests that students’ perceptions of support services were connected with several other dimensions of their institutional experience. Given that SSS, AQSCP, and PLE were measured from the same respondents at the same point in time, the magnitude of the larger coefficients involving SSS should be interpreted cautiously because some shared method variance cannot be excluded. However, the proposed relationship between the learning environment and academic quality and preparedness (Hypothesis 4–2) was not statistically supported, indicating that physical infrastructure alone may not be sufficient to shape students’ perceptions of educational quality and preparedness. These structural relationships should be understood as associations within the specified cross-sectional model rather than as evidence of causal effects. Figure 3 presents the final structural model results.

Indirect effect analysis, which is presented in Table 3, revealed no statistically significant indirect pathways from the selected background variables to PLE. Specifically, none of the proposed indirect pathways involving gender, funding type, or GPA yielded statistically significant effects. The absence of significant indirect effects is consistent with the limited direct associations observed between the selected background characteristics and PLE. Within the tested model, SSS and AQSCP showed significant direct associations with PLE, whereas the selected background characteristics showed no significant direct or indirect associations with PLE. A weak but significant positive correlation was observed between GPA and funding type (*β* = 0.17), and a weak association between prior GPA and funding type. Given the cross-sectional design and categorical nature of funding status, this correlation should not be interpreted as evidence that academic performance determines scholarship receipt. These comparisons should nevertheless be interpreted cautiously given the preliminary measurement structure and cross-sectional design.

**Table 3:** Results of indirect path analysis.

Parameter	Estimate	*p*-value	Interpretation
Indirect paths’ effect on students’ PLE			
Funding type → AAR → PLE	0.012	0.093	H5i: Rejected
Funding type → LEI → PLE	0.008	0.655	H5ii: Rejected
Funding type → AQSCP → PLE	−0.035	0.149	H5iii: Rejected
Funding type → LEI → AQSCP → PLE	0.018	0.24	H5iv: Rejected
Gender → LEI → PLE	0.003	0.694	H6i: Rejected
Gender → LEI → AQSCP → PLE	0.008	0.438	H6ii: Rejected
GPA → LEI → PLE	−0.007	0.562	H7i: Rejected
GPA → AQSCP → PLE	−0.019	0.799	H7ii: Rejected
GPA → SSS → PLE	−0.016	0.219	H7iii: Rejected
GPA → SSS → LEI → PLE	0.001	0.886	H7iv: Rejected
GPA → SSS → LEI → AQSCP → PLE	−0.008	0.487	H7v: Rejected
GPA → LEI → AQSCP → PLE	−0.02	0.237	H7vi: Rejected
Total indirect effects on students’ PLE			
Funding type	0.004	0.948	No significant indirect effect on learning effectiveness
Gender	0.012	0.486	
Prior Education GPA	−0.069	0.397	

## 4 Discussion

This study examined how selected student background characteristics and post-enrollment academic support conditions relate to international students’ perceived learning effectiveness in Chinese higher education. By integrating SERVQUAL-informed perceived academic support dimensions with Kirkpatrick’s learning-evaluation perspective, the study developed a framework for examining how support services, communication, learning conditions, academic quality, safety, and career preparedness are associated with students’ perceived learning development. Within the tested model, SSS and AQSCP were significantly associated with PLE, whereas the selected background characteristics showed no significant direct associations. This pattern directs attention to post-enrollment support conditions without establishing that these conditions are more important than student background characteristics.

First, the non-significant direct and indirect pathways for gender and prior academic performance indicate that this analysis did not detect statistically significant associations between these variables and PLE in this sample. These findings should not be interpreted as evidence that gender or prior academic performance has no role in international students’ learning more generally or that student groups are equivalent. Rather, the results support considering institutional support conditions alongside student characteristics, rather than treating background characteristics as sufficient explanations of perceived learning effectiveness. This result is broadly consistent with Hyun (2019) and Qu and Cross (2024), who emphasized the role of institutional environments in shaping international students’ educational experiences.

Funding type was associated with perceptions of academic and administrative reliability and learning environment quality, but not directly with PLE. Because university tier, departmental resources, and scholarship-management procedures were not measured, the mechanisms underlying these associations cannot be determined. The findings therefore indicate differences in perceived institutional conditions associated with funding status within this sample, rather than demonstrating that funding arrangements themselves create unequal support environments. This interpretation is broadly consistent with Qu and Cross (2024), who showed that scholarship-supported students may experience stronger institutional support systems.

Second, student support services showed the broadest pattern of significant associations among the institutional dimensions examined in the tested model. Student support services were positively associated with perceived learning effectiveness and with academic and administrative reliability, learning environment, academic quality, safety, and career preparedness. This pattern suggests that students’ perceptions of support services covaried with several other aspects of their institutional experience. One possible interpretation is that accessible support may be connected to students’ confidence in navigating academic expectations and communicating with institutional actors, consistent with Marlina’s (2009) emphasis *on* the importance of responsive institutional support for international students’ academic engagement and participation. It also aligns with educational psychology research showing that perceived support is related to students’ engagement, learning attitudes, and anxiety (Zhou et al., 2025). However, these psychological and behavioral pathways were not measured directly in the present study and should be examined in future research.

Academic and administrative reliability and the learning environment were not significantly associated with PLE when the other institutional dimensions were included in the model. This finding should not be interpreted as evidence that facilities, procedures, or infrastructure are unimportant. It indicates only that the specified model did not detect independent direct associations with PLE. Whether these conditions contribute indirectly, interact with other forms of support, or operate differently across institutions requires further investigation.

Third, the findings contribute to research on international student learning in non-Anglophone higher education contexts. China and similar host systems have expanded international student recruitment, yet post-enrollment support remains essential for ensuring that internationalization leads to meaningful learning experiences. While language barriers, academic communication, administrative navigation, and social integration are often discussed as student adaptation challenges, the present findings suggest that they are also institutional design issues. Universities need to consider how academic support, advising, communication channels, counseling access, and career-preparation structures are organized for students studying in unfamiliar educational and linguistic environments.

## 5 Implications and future research

### 5.1 Theoretical contributions

This study provides a complementary perspective on international student learning by linking institutional support conditions with perceived learning effectiveness in a non-Anglophone higher education context. Rather than treating international students’ learning experiences mainly as outcomes of individual background characteristics, the study integrates background variables, post-enrollment academic support conditions, and perceived learning effectiveness within one analytical framework. This integration extends existing research on international student adaptation by showing that perceived learning effectiveness can be examined in relation to the institutional conditions students encounter after enrollment.

The study also clarifies the role of student support services within the wider learning environment. The findings suggest that support services are not only administrative or welfare-related functions. They may operate as a connecting institutional condition that is associated with the students’ perceptions of academic and administrative reliability, learning environment quality, academic quality, safety, career preparedness, and perceived learning effectiveness. This finding adds evidence to educational psychology and international higher education research by showing that students’ perceptions of SSS were associated with several other institutional dimensions and with PLE. The present analysis did not test whether academic participation, communication access, confidence, or institutional navigation mediate these relationships; these remain potential pathways for future investigation.

Finally, the study contributes to framework development by connecting institutional support conditions with a broader conception of perceived educational effectiveness. SERVQUAL-informed dimensions identify the post-enrollment conditions through which international students encounter academic and administrative reliability, support, learning resources, safety, and career preparation, while the Kirkpatrick-informed perspective extends the outcome beyond satisfaction to include perceived learning, application, confidence, and future preparedness. The contribution of this integration lies in distinguishing the institutional conditions students experience from how they evaluate the educational consequences of those conditions and examining the relationship between the two within one analytical framework. This approach is particularly relevant to international higher education because students must navigate academic, administrative, linguistic, and support environments that may be unfamiliar to them. The framework does not assume a causal sequence among the Kirkpatrick-inspired categories and does not replace objective learning assessment; rather, it provides a context-sensitive structure for examining how perceived institutional support is associated with students’ perceived learning effectiveness.

### 5.2 Implications for academic support and institutional practice

The findings have practical implications for how universities evaluate and improve international students’ post-enrollment learning experiences. Because student support services and academic quality, safety, and career preparedness were the institutional dimensions showing significant associations with perceived learning effectiveness in the tested model, universities may consider examining students’ access to academic advising, communication support, counseling, career-related guidance, and other forms of post-enrollment support when evaluating international student experiences. The present data do not establish that expanding these services will improve PLE; such effects require longitudinal or intervention-based research.

The non-significant paths involving selected background characteristics, academic and administrative reliability, and learning environment should likewise not be interpreted as evidence that these factors are unimportant. Rather, independent direct associations with PLE were not detected for these variables within the specified model. Universities may therefore continue to evaluate infrastructure, administrative clarity, and background-related support needs while also examining whether academic and support services are accessible and coordinated. The associations between SSS and several other perceived institutional dimensions provide a rationale for future research testing whether more coordinated support provision improves students’ ability to locate assistance and navigate academic expectations.

These implications may also be relevant to other non-Anglophone higher education systems facing similar language, communication, and adaptation challenges, but they should not be generalized beyond the present context without further evidence. Comparative, longitudinal, and multi-institutional studies are needed to determine whether the observed associations hold across different institutional and national settings.

### 5.3 Limitations and future research

Several limitations should be recognized. First, although the sample included international students from multiple nationalities, academic levels, and Chinese higher education institutions, the final sample size was modest relative to the diversity of the international student population in China. The sample was also unevenly distributed across nationalities, institutions, and funding categories. Therefore, the findings should not be interpreted as statistically representative of all international students in China. Rather, the study should be understood as an exploratory, context-sensitive analysis of how perceived academic support dimensions relate to perceived learning effectiveness among the surveyed students. Future research should use larger and more stratified samples across university type, region, discipline, funding category, academic level, and country of origin.

Given that support services, academic quality, safety, and career preparedness, and PLE were measured from the same respondents at the same point in time, the magnitude of the larger coefficients involving support services should be interpreted cautiously because some shared method variance cannot be excluded.

Second, the study relied on self-reported data collected from the same respondents at a single point in time. This design was appropriate for examining students’ perceptions of institutional support and perceived learning effectiveness, but it also creates the possibility of common method variance because the focal constructs were measured within the same survey context. The questionnaire was anonymous, which may have reduced evaluation apprehension and social desirability, but anonymity cannot eliminate shared method effects. Although the post-hoc single-factor CFA indicated that one dominant factor did not adequately account for the covariance among the measures, partial common method variance cannot be ruled out. Some of the larger associations, particularly those involving SSS, AQSCP, and PLE, should therefore be interpreted cautiously and may partly reflect shared method effects. Self-report may also be affected by recall error and differences in item interpretation. Future studies should use temporally separated or multi-source measurement and combine student-reported perceptions with administrative records, institutional service-use data, learning analytics, and objective academic outcomes.

Third, perceived learning effectiveness was measured as students’ self-evaluations of their learning experience, not as objective academic achievement or longitudinal learning gains. This distinction is important because perceived learning effectiveness captures students’ academic confidence, communication improvement, knowledge application, and future preparedness, but it does not directly measure grades, completion outcomes, or long-term academic development. Future studies should examine whether perceived learning effectiveness is associated with objective academic performance, retention, degree completion, or post-graduation outcomes.

Finally, the measurement and structural models demonstrated acceptable absolute and approximate fit but marginal incremental fit. In addition, the EFA and follow-up CFA were conducted using the same analytical sample because the available sample size did not support separate development and validation subsamples. The proposed factor structure should therefore be regarded as preliminary rather than independently confirmed, and the structural coefficients should be interpreted as associations conditional on this measurement specification rather than as definitive or causal effects. More broadly, non-significant paths should not be interpreted as evidence that no relationship exists, and differences in the apparent magnitude of coefficients across background and institutional predictors were not formally tested as pairwise coefficient differences. The modification-index analysis also indicated localized overlap among some support, cultural-integration, and learning-related indicators. In particular, PLE was modeled as one overarching construct using 13 indicators spanning conceptually related aspects of Reaction, Learning, Behavior, and Results. Although these levels were used as conceptual categories rather than separate subscales, some residual multidimensionality may remain and may have contributed to the marginal incremental fit. Future research should replicate the measurement structure using a larger independent sample and compare the present overarching PLE specification with alternative multidimensional or higher-order models. Longitudinal or mixed-methods designs would also help determine how international students’ support needs and learning perceptions change across different stages of study.

## Data Availability

The datasets presented in this article are not readily available because they contain participant-level survey responses and are subject to ethical and privacy restrictions under the approved research protocol. Requests to access the anonymized datasets should be directed to the corresponding author.
