# Supplementary material for: Post-enrollment academic support and perceived learning effectiveness among international students in Chinese universities
Source: Front Psychol. 2026 Sep 30;17:1937334. doi: 10.3389/fpsyg.2026.1937334 (PMC13662131; doi:10.3389/fpsyg.2026.1937334)
Supplement: Supplementary file 1 [file Supplementary_file_1.DOCX]

Supplementary Material

**Supplementary Table 1.** Institutional Service Dimension (ISD) Framework

| SERQUAL Levels | Code | Indicators | Sources |
| --- | --- | --- | --- |
| Physical & Technological Infrastructure (Tangibles) | T1 | Lighting in Lecture/Seminar Rooms | (Hoque et al., 2023) |
|  | T2 | Comfortable Room Temperature | (Hoque et al., 2023) |
|  | T3 | Modern and Up-to-date Equipment | (Brochado, 2009; Yousapronpaiboon, 2014; Douglas et al., 2006; Hoque et al., 2023) |
|  | T4 | Buildings'Accessibility | (Brochado, 2009; Hoque et al., 2023;Yousapronpaiboon, 2014) |
|  | T5 | Visual Appeal of Materials | (Yousapronpaiboon, 2014) |
|  | T6 | Convenient Operating Hours | (Yousapronpaiboon, 2014) |
| Academic & Administrative Consistency (Reliability) | Rl1 | Registration Efﬁciency | (Hoque et al., 2023) |
|  | Rl2 | Accuracy of Student Records | (Brochado, 2009; Hoque et al., 2023) |
|  | Rl3 | Regularity of Lectures and Seminars | (Hoque et al., 2023) |
|  | Rl4 | Timeliness of University Services | (Brochado, 2009; Hoque et al., 2023) |
|  | Rl5 | Reliability of Promised Services | (Yousapronpaiboon, 2014) |
|  | Rl6 | Manageable course workload | (Douglas et al., 2006) |
|  | Rl7 | Adequacy of Chinese Language Course in Helping IS' Communication | Added based on the study's goals. |
|  | Rl8 | Appropriateness of Assessment Methods | (Douglas et al., 2006) |
| Student Support & Communicatio（Responsiveness) | Rs1 | Availability of Help for Students | (Hoque et al., 2023) |
|  | Rs2 | Administrative Staff Problem-Solving Capacity | (Hoque et al., 2023; Yousapronpaiboon, 2014) |
|  | Rs3 | Promptness of Service | (Yousapronpaiboon, 2014) |
|  | Rs4 | Communication Channels for Complaints | (Hoque et al., 2023) |
|  | Rs5 | Availability of Lecturers for Consultation | (Douglas et al., 2006; Hoque et al., 2023) |
|  | Rs6 | Academicians Promptness Response | (Hoque et al., 2023) |
|  | Rs7 | Language Support | Added based on the study's goals. |
|  | Rs8 | Medical Services | Added based on the study's goals. |
|  | Rs9 | Counseling Services | (Abdullah, 2006) |
|  | Rs10 | Communication of Available Resources and Support Programs | Added based on the study's goals. |
| Faculty Competence & Credibility (Assurance) | A1 | Qualifications and Experience of Academicians | (Abdullah, 2006b) |
|  | A2 | Academicians' Knowledge and Effectiveness | (Douglas et al., 2006; Yousapronpaiboon, 2014) |
|  | A3 | Educators' Proficiency in Teaching and Research | (Abdullah, 2006; Douglas et al., 2006; Hoque et al., 2023) |
|  | A4 | Academicians' Interaction with Students | (Douglas et al., 2006; Hoque et al., 2023) |
|  | A5 | Trust in University Staff | (Brochado, 2009; Yousapronpaiboon, 2014) |
|  | A6 | Management Focus on Students' Needs | (Hoque et al., 2023) |
|  | A7 | University Safety and Security Measures | (Hoque et al., 2023) |
|  | A8 | Assignment & Exam Criteria Clarity | (Douglas et al., 2006) |
|  | A9 | University Reputation | Added based on the study's goals. |
|  | A10 | Cultural Awareness Training | Added based on the study's goals. |
|  | A11 | Job Market Alignment | Added based on the study's goals. |
| Student-Centred Services (Empathy) | E1 | Staff Understanding of Students' Needs | (Yousapronpaiboon, 2014) |
|  | E2 | Cultural Integration Programs | Added based on the study's goals. |
|  | E3 | Supportive Student Affairs | Added based on the study's goals. |
|  | E4 | Interaction Between Local and IS | Added based on the study's goals. |
|  | E5 | IS' Daily Life Assistance | Added based on the study's goals. |

**Supplementary Table 2.** PLE indicators and Kirkpatrick-inspired conceptual grouping

| Conceptual grouping | PLE indicator |
| --- | --- |
| *Reaction* | Teaching Methods Engagement and Effectiveness |
|  | Overall Educational Experience Satisfaction |
|  | Satisfaction with Cultural Integration Programs |
| *Learning* | Knowledge Gained in Major Field |
|  | Understanding of Chinese Culture |
|  | Practical Skills Development |
| *Behavior* | Application of Knowledge and Skills |
|  | Effectiveness in Diverse Collaboration |
|  | Communication and Collaboration Improvement |
|  | Confidence in Applying Learning |
| *Results* | Professional Networking and Integration Opportunities |
|  | Preparation for Career or Further Studies |
|  | Academic Performance Improvement |

*Note: The four Kirkpatrick-inspired categories were used to organize the content of the 13 PLE indicators. They were not specified as separate latent subscales in the CFA or SEM; the 13 indicators loaded on one overarching PLE construct.*

**Supplementary Table 3.** Exploratory Kruskal–Wallis analysis of ISD indicators across demographic variables

| ISD Indicators | Gender | Funding Type | Previous Education Level GPA | Previous Education Country | English Proficiency | Chinese Proficiency |
| --- | --- | --- | --- | --- | --- | --- |
| T1 | 6.46* | 10.43 | 5.08 | 8.34 | 6.52 | 1.74 |
| T2 | 2.26 | 12.32* | 9.7* | 1.90 | 2.00 | 2.24 |
| T3 | 0.40 | 9.02 | 8.69* | 5.03 | 1.68 | 1.22 |
| T4 | 5.61 | 17.01** | 5.36 | 9.71 | 1.62 | 2.27 |
| T5 | 10.38** | 9.19 | 5.60 | 18.7** | 2.25 | 0.63 |
| T6 | 5.53 | 9.34 | 0.59 | 7.32 | 0.95 | 1.96 |
| Rl1 | 5.13 | 8.67 | 4.55 | 3.13 | 0.81 | 0.41 |
| Rl2 | 0.67 | 15.91** | 3.35 | 6.37 | 11.5** | 3.46 |
| Rl3 | 6.44* | 6.22 | 4.52 | 4.40 | 1.68 | 4.26 |
| Rl4 | 4.62 | 8.98 | 0.22 | 15.55** | 6.93 | 6.82 |
| Rl5 | 2.30 | 4.66 | 8.06* | 3.94 | 3.30 | 2.08 |
| Rl6 | 6.03* | 7.79 | 6.83 | 9.28 | 2.01 | 0.99 |
| Rl7 | 4.37 | 7.84 | 1.65 | 6.16 | 3.49 | 21.69** |
| Rl8 | 0.75 | 4.51 | 1.61 | 1.58 | 2.95 | 5.55 |
| Rs1 | 1.74 | 5.10 | 3.90 | 5.19 | 7.39 | 1.07 |
| Rs2 | 1.79 | 7.82 | 1.82 | 6.31 | 8.48 | 5.96 |
| Rs3 | 1.48 | 2.12 | 2.09 | 4.63 | 2.47 | 2.40 |
| Rs4 | 4.31 | 5.12 | 1.55 | 8.03 | 2.94 | 6.44 |
| Rs5 | 0.09 | 5.70 | 2.44 | 2.83 | 6.59 | 4.45 |
| Rs6 | 3.10 | 2.80 | 2.84 | 2.95 | 0.42 | 5.40 |
| Rs7 | 5.99* | 6.02 | 3.93 | 8.54 | 3.61 | 7.58 |
| Rs8 | 13.48** | 3.53 | 7.19 | 10.59 | 3.40 | 0.45 |
| Rs9 | 6.46* | 3.49 | 1.88 | 2.95 | 7.55 | 0.94 |
| Rs10 | 4.92 | 4.13 | 2.60 | 4.28 | 2.47 | 0.50 |
| A1 | 0.25 | 10.65 | 1.53 | 3.08 | 6.07 | 1.34 |
| A2 | 0.16 | 10.59 | 3.50 | 2.30 | 6.02 | 2.78 |
| A3 | 0.19 | 16.2** | 2.30 | 6.47 | 3.11 | 1.29 |
| A4 | 2.25 | 4.90 | 1.87 | 5.18 | 3.12 | 3.02 |
| A5 | 0.94 | 9.87 | 6.31 | 5.19 | 2.60 | 4.59 |
| A6 | 5.33 | 5.65 | 5.33 | 1.25 | 8.27 | 6.05 |
| A7 | 8.95* | 7.11 | 7.78 | 3.34 | 3.06 | 2.66 |
| A8 | 3.20 | 2.92 | 6.01 | 2.60 | 5.76 | 1.72 |
| A9 | 0.97 | 3.78 | 5.81 | 8.27 | 5.10 | 1.67 |
| A10 | 0.42 | 9.04 | 3.70 | 5.81 | 4.72 | 0.63 |
| A11 | 1.13 | 4.88 | 1.18 | 12.85* | 0.25 | 1.85 |
| E1 | 1.34 | 6.61 | 7.72 | 3.25 | 1.49 | 1.39 |
| E2 | 0.79 | 9.56 | 3.23 | 1.53 | 2.77 | 1.57 |
| E3 | 0.62 | 3.18 | 4.06 | 4.97 | 5.65 | 4.32 |
| E4 | 1.33 | 11.41 | 0.79 | 3.75 | 7.27 | 1.73 |
| E5 | 2.51 | 3.5* | 10.57* | 4.33 | 3.13 | 2.25 |

*Note: * denotes p-value < 0.05; ** denotes p-value < 0.01*

**Supplementary Table 4.** Measurement and structural model fit indices

| Fit index | Measurement model | Structural model | Single-factor CFA (CMB diagnostic) | Reference criterion |
| --- | --- | --- | --- | --- |
| CMIN/DF | 1.737 | 1.766 | 3.72 | < 2.0 good; < 3.0 acceptable |
| RMSEA | .058 | .059 | .112 | < .06 good; < .08 acceptable |
| RMR | .041 | .041 | — | < .05 |
| CFI | .892 | .885 | .629 | ≥ .90 |
| TLI | .870 | .865 | .602 | ≥ .90 |

*Note: The single-factor CFA was conducted as a post-hoc diagnostic of potential common method variance by loading all 30 retained ISD and PLE indicators onto one latent factor. Its poor fit indicates that a single dominant common factor does not adequately account for the covariance among the self-reported measures. This diagnostic does not rule out partial common method variance.*

**Supplementary Table 5.** Measurement-model residual covariance and MI diagnostics

| Suggested parameter | MI | EPC | Construct relationship | Decision |
| --- | --- | --- | --- | --- |
| *Counseling Services ↔ Local–International Student Interaction* | 15.678 | .135 | SSS–AQSCP | Not added |
| *Job Market Alignment ↔ Satisfaction with Cultural Integration Programs* | 15.461 | .116 | AQSCP–PLE | Not added |
| *Satisfaction with Cultural Integration Programs ↔ SSS* | 12.666 | .099 | PLE–SSS | Not added |
| *Academic Performance Improvement ↔ SSS* | 11.437 | −.071 | PLE–SSS | Not added |
| *Adequacy of Chinese Language Course ↔ Understanding of Chinese Culture* | 11.415 | .103 | SSS–PLE | Not added |
| *Adequacy of Chinese Language Course ↔ Effectiveness in Diverse Collaboration* | 10.569 | −.076 | SSS–PLE | Not added |
| *Reliability of Promised Services ↔ Effectiveness in Diverse Collaboration* | 10.564 | .063 | AAR–PLE | Not added |
| *Accuracy of Student Records ↔ Practical Skills Development* | 10.332 | −.065 | AAR–PLE | Not added |

*Note: MI = modification index; EPC = expected parameter change. All modification indices were inspected; only remaining covariance MIs ≥ 10 are shown. No suggested additional regression weight or cross-loading reached MI ≥ 10; the largest was MI = 7.441. Cross-construct parameters were not added solely to improve global model fit.*

**Supplementary Table 6.** Results of indirect path analysis

| Indirect paths' effect on students' PLE | | | |
| --- | --- | --- | --- |
| Parameter | **Estimate** | **P-value** | **Interpretation** |
| *Funding type ---> AAR---> PLE* | 0.012 | 0.093 | H5i: Rejected |
| *Funding type --->* LEI *---> PLE* | 0.008 | 0.655 | H5ii: Rejected |
| *Funding type ---> AQSCP ---> PLE* | -0.035 | 0.149 | H5iii: Rejected |
| *Funding type --->* LEI *---> AQSCP ---> PLE* | 0.018 | 0.24 | H5iv: Rejected |
| *Gender --->* LEI *---> PLE* | 0.003 | 0.694 | H6i: Rejected |
| *Gender --->* LEI *--->* *AQSCP ---> PLE* | 0.008 | 0.438 | H6ii: Rejected |
| *GPA --->* LEI *---> PLE* | -0.007 | 0.562 | H7i: Rejected |
| *GPA ---> AQSCP ---> PLE* | -0.019 | 0.799 | H7iii: Rejected |
| *GPA ---> SSS ---> PLE* | -0.016 | 0.219 | H7iii: Rejected |
| *GPA ---> SSS --->* LEI *---> PLE* | 0.001 | 0.886 | H7iv: Rejected |
| *GPA --->* *SSS --->* LEI *---> AQSCP ---> PLE* | -0.008 | 0.487 | H7v: Rejected |
| *GPA* *--->* LEI *---> AQSCP ---> PLE* | -0.02 | 0.237 | H7vi: Rejected |
| Total indirect effects on students' PLE | | | |
| Parameter | **Estimate** | **P-value** | **Interpretation** |
| *Funding type* | 0.004 | 0.948 | No significant indirect effect on learning effectiveness |
| *Gender* | 0.012 | 0.486 |  |
| *Prior Education GPA* | -0.069 | 0.397 |  |

**Supplementary Table 7.** Additional reliability and discriminant-validity evidence for the ISD measurement model

**Panel A**. Standardized CFA loadings and composite reliability

| Construct | Item | Standardized CFA loading | CR |
| --- | --- | --- | --- |
| AAR | Rl4 | .739 | .803 |
|  | Rl3 | .696 |  |
|  | Rl2 | .770 |  |
|  | Rl5 | .632 |  |
| SSS | Rs7 | .682 | .722 |
|  | Rl7 | .577 |  |
|  | Rs9 | .698 |  |
|  | Rl6 | .549 |  |
| LEI | T2 | .528 | .767 |
|  | T3 | .597 |  |
|  | T1 | .525 |  |
|  | T4 | .714 |  |
|  | T5 | .770 |  |
| AQSCP | A3 | .590 | .678 |
|  | A7 | .545 |  |
|  | A11 | .569 |  |
|  | E4 | .643 |  |

**Panel B.** HTMT matrix

|  | AAR | SSS | LEI | AQSCP |
| --- | --- | --- | --- | --- |
| AAR | — |  |  |  |
| SSS | .569 | — |  |  |
| LEI | .668 | .567 | — |  |
| AQSCP | .670 | .638 | .578 | — |

*Note: CR = composite reliability; HTMT = heterotrait–monotrait ratio. All standardized CFA loadings exceeded .50. HTMT values are reported relative to commonly used .85 and .90 criteria.*
